# Supplementary material for: Male sex pheromone components in Heliconius butterflies released by the androconia affect female choice
Source: PeerJ. 2017 Nov 7;5:e3953. doi: 10.7717/peerj.3953 (PMC5680698; doi:10.7717/peerj.3953)
Supplement: Supplemental Information 5 [file peerj-05-3953-s005.docx]

**Supplementary**

Table S1: Comparison of amount of each compound in different wing areas of males (10 days post-eclosion, Ecuador samples). FWover refers to the overlapping region of the forewing, FWrest, the remaining portion of the wing, HWand, the hindwing androconial overlapping region, and HWrest, the remaining portion of the wing. Results of Kruskal-Wallis reported for each compound and of post-hoc analysis (Dunn test), where * signifies <0.05, ** <0.01 and *** <0.001.

|  |  |  |  | Difference in mean amount of compound between areas | | | | | |
| --- | --- | --- | --- | --- | --- | --- | --- | --- | --- |
| Compound | x^2^ | df | p | FWrest-FWover | HWand-FWover | HWrest-FWover | HWand-FWrest | HWrest-FWrest | HWrest-HWand |
| (*Z*)-9-octadecenal | 6.9 | 3 | NS | -0.228 | 1.586 | -0.228 | 1.814 | -0.000 | -1.814 |
| octadecanal | 16.8 | 3 | <0.001 | -10.95 | 8.40 | -12.43 | 19.35* | -1.48 | -20.83** |
| (*Z*)-11-icosenal | 16.8 | 3 | <0.001 | -4.05 | 2.65 | -4.05 | 6.70** | 0.00 | -6.70** |
| icosanal | 15.1 | 3 | <0.01 | -0.50 | 1.38 | -0.50 | 1.88** | 0.00 | -1.88** |
| (*Z*)-13-docosenal | 14.5 | 3 | <0.01 | -1.45 | 1.31 | -1.45 | 2.76* | 0.00 | -2.76* |

Table S2: Comparison of amount of each compound in different sex and age categories (mature individuals being 10 days post-eclosion and young 0 days post-eclosion, Ecuador samples). Results of Kruskal-Wallis reported for each compound and of post-hoc analysis (Dunn test), where * signifies <0.05, ** <0.01 and *** <0.001.

|  |  |  |  | Difference in mean amount of compound between categories | | |
| --- | --- | --- | --- | --- | --- | --- |
| Compound | x^2^ | df | p | Mature female - Young male | Mature male - Young male | Mature male -Young female |
| (*Z*)-9-octadecenal | 6.9 | 2 | <0.05 | 0.00 | 1.81 | 1.81 |
| octadecanal | 13.3 | 2 | <0.01 | 0.00 | 20.83** | 20.83** |
| (*Z*)-11-icosenal | 13.3 | 2 | <0.01 | 0.00 | 6.70** | 6.70** |
| icosanal | 13.3 | 2 | <0.01 | 0.00 | 1.88** | 1.88** |
| (*Z*)-13-docosenal | 13.3 | 2 | <0.01 | 0.00 | 2.76** | 2.76** |
| henicosane | 4.4 | 2 | NS | -8.13 | 4.30 | 12.43 |

Table S3: Compounds identified in *H. melpomene rosina* (Panama samples) with a mean amount greater than 1 ng in at least one of the sexes. Mean amounts ± standard deviation listed for both male and female butterflies (10-12 days post-eclosion), as well as the percentage of individuals in which the compound was detected. Kruskal-Wallis non-parametric test p-values highlight differences between males and females. Compounds identified by comparison with synthetic samples are shown in bold. Potential contaminants are denoted with an asterisk.

| Chemical | Kovats RI | Male (ng) | % | Female | % |  |
| --- | --- | --- | --- | --- | --- | --- |
| ***o*-Guaiacol** | 1090 | 1.5 ± 1.7 | 75 | 0.1 ± 0.2 | 18 | <0.01 |
| **Nonanal** | 1105 | 24.7 ± 34.2 | 100 | 1.5 ± 1.7 | 73 | <0.001 |
| **Benzoic acid** | 1166 | 2.6 ± 6.7 | 25 | 0 ± 0 | 0 | NS |
| **Naphthalene*** | 1181 | 1.2 ± 1.1 | 85 | 1.0 ± 1.6 | 36 | NS |
| Decanal | 1198 | 1.7 ± 5.5 | 30 | 0.2 ± 0.7 | 9 | NS |
| **Ethyl 4-ethoxybenzoate** | 1527 | 7.0 ± 5.1 | 95 | 0.7 ± 1.2 | 27 | <0.001 |
| Dihydroactinidiolide | 1532 | 1.1 ± 1.6 | 60 | 0 ± 0 | 0 | <0.01 |
| **Syringaldehyde** | 1662 | 649.9 ± 398.1 | 100 | 0 ± 0 | 0 | <0.001 |
| 3,5-Dimethoxy-4-hydroxybenzyl alcohol | 1707 | 4.0 ± 6.9 | 50 | 0 ± 0 | 0 | <0.01 |
| Unknown hydrocarbon | 1709 | 1.1 ± 1.6 | 50 | 0.3 ± 0.6 | 18 | NS |
| 1-(3,5-Dimethoxy-4-hydroxybenzyl)ethanone | 1735 | 1.3 ± 2.0 | 60 | 0 ± 0 | 0 | <0.01 |
| Unknown aromatic compound | 1738 | 5.5 ± 5.5 | 75 | 0 ± 0 | 0 | <0.001 |
| Unknown aromatic compound | 1757 | 2.1 ± 2.8 | 55 | 0 ± 0 | 0 | <0.01 |
| Ethyl benzoate | 1762 | 1.5 ± 1.4 | 80 | 1.3 ± 1.3 | 73 | NS |
| Methyl 1*H*-indol-3-acetate | 1822 | 3.2 ± 3.2 | 85 | 0 ± 0 | 0 | <0.001 |
| Unknown compound | 1828 | 0.0 ± 0.1 | 5 | 2.1 ± 7.0 | 9 | NS |
| Methyl 1*H*-indol-3-carboxylate | 1853 | 2.6 ± 2.3 | 90 | 0 ± 0 | 0 | <0.001 |
| Unknown compound | 1914 | 1.2 ± 1.8 | 45 | 0.2 ± 0.4 | 27 | NS |
| Unknown aldehyde | 1968 | 6.9 ± 11.0 | 35 | 0 ± 0 | 0 | <0.05 |
| Unknown compound | 1981 | 0.0 ± 0.0 | 0 | 1.2 ± 3.9 | 9 | NS |
| **(*Z*)-9-Octadecenal** | 1996 | 12.1 ± 14.0 | 80 | 0 ± 0 | 0 | <0.001 |
| **Octadecanal** | 2021 | 740.7 ± 411.4 | 100 | 0 ± 0 | 0 | <0.001 |
| Unknown compound | 2053 | 2.0 ± 8.8 | 80 | 0 ± 0 | 0 | NS |
| Ethyl 4-hydroxy-3,5- dimethoxybenzoate | 2057 | 41.6 ± 67.5 | 80 | 0 ± 0 | 0 | <0.001 |
| Unknown compound | 2064 | 2.9 ± 5.0 | 30 | 0 ± 0 | 0 | <0.05 |
| Methyloctadecanal | 2065 | 6.5 ± 6.5 | 65 | 0 ± 0 | 0 | <0.001 |
| Henicosadiene | 2065 | 1.3 ± 4.2 | 10 | 0 ± 0 | 0 | NS |
| Methyloctadecanal | 2072 | 4.9 ± 3.7 | 80 | 0 ± 0 | 0 | <0.001 |
| Methyloctadecanal | 2077 | 24.4 ± 20.9 | 65 | 0 ± 0 | 0 | <0.001 |
| Unknown compound | 2078 | 5.5 ± 24.4 | 5 | 0 ± 0 | 0 | NS |
| **Octadecan-1-ol** | 2082 | 232.5 ± 149.7 | 100 | 0 ± 0 | 0 | <0.001 |
| **Henicosane** | 2099 | 8.7 ± 16.2 | 65 | 0.7 ± 2.5 | 9 | <0.01 |
| Unknown alkene or alcohol | 2127 | 16.3 ± 8.2 | 90 | 0 ± 0 | 0 | <0.001 |
| Unknown compound | 2132 | 35.3 ± 22.9 | 80 | 0 ± 0 | 0 | <0.001 |
| Unknown compound | 2137 | 6.2 ± 13.1 | 25 | 0 ± 0 | 0 | NS |
| Methyloctadecan-1-ol | 2138 | 7.3 ± 7.3 | 55 | 0 ± 0 | 0 | <0.01 |
| Unknown hydrocarbon | 2143 | 4.0 ± 2.4 | 45 | 0 ± 0 | 0 | NS |
| Oleyl acetate | 2181 | 2.0 ± 8.9 | 5 | 0 ± 0 | 0 | NS |
| **(*Z*)-11-Icosenal** | 2198 | 164.0 ± 127.0 | 100 | 0 ± 0 | 0 | <0.001 |
| **Icosanal** | 2224 | 22.8 ± 11.3 | 100 | 0 ± 0 | 0 | <0.001 |
| **(*Z*)-11-Icosenol** | 2261 | 615.7 ± 304.0 | 100 | 0 ± 0 | 0 | <0.001 |
| Tricosene | 2281 | 2.4 ± 2.1 | 75 | 0 ± 0 | 0 | <0.001 |
| Unknown compound | 2290 | 1.9 ± 3.5 | 30 | 0 ± 0 | 0 | <0.05 |
| **Tricosane** | 2297 | 1.0 ± 2.4 | 40 | 3.2  ± 4.3 | 45 | NS |
| Unknown compound | 2352 | 1.9 ± 5.7 | 15 | 0 ± 0 | 0 | NS |
| Unknown compound | 2392 | 11.8 ± 18.3 | 70 | 6.8  ± 7.8 | 73 | NS |
| **(*Z*)-13-Docosenal** | 2403 | 64.2 ± 54.6 | 90 | 0 ± 0 | 0 | <0.001 |
| Unknown compound | 2459 | 19.9 ± 20.6 | 65 | 0 ± 0 | 0 | <0.001 |
| **(*Z*)-13-Docosen-1-ol** | 2464 | 10.1 ± 23.5 | 20 | 0 ± 0 | 0 | NS |
| Unknown compound | 2465 | 3.3 ± 8.0 | 20 | 0 ± 0 | 0 | NS |
| Unknown compound | 2498 | 0.2 ± 0.7 | 5 | 5.8 ± 13.5 | 18 | NS |
| **Pentacosane** | 2500 | 3.1 ± 4.7 | 55 | 37.6 ± 38.4 | 82 | <0.01 |
| 11-Methylpentacosane | 2533 | 0.0 ± 0 | 0 | 105.9 ±  111.3 | 100 | <0.001 |

Figure S1: NMDS in three dimensions on chemical compounds of male hindwings of *H. melpomene* soaked for 1 or 3 hours in solvent (10-12 days post-eclosion, Panama samples). Stress=0.03. Each different colour corresponds to an individual. Closed circles are from the 1-hour group, and open squares 3-hours. No compounds were found in significantly different amounts between groups (Paired t-test, df=4, p=NS).

Figure S2: NMDS in three dimensions on chemical compounds of male and female wings of *H. melpomene* (10-12 days post-eclosion, Panama samples). Stress=0.05. Differences between males and females was supported by ANOSIM (Bray-Curtis R-statistic=0.9226, p=0.001)

Figure S3: Difference in number of courtships by control and experimental males represented by boxplots. Each point represents an experiment. Difference not significantly different from zero for *H. erato demophoon*, *H. melpomene* *rosina* and *H. melpomene malleti*. Control *H. timareta florencia* males courted more than experimental males, complicating the interpretation in this species (Wilcoxin Signed-Rank Test, V=138.5, p<0.01).


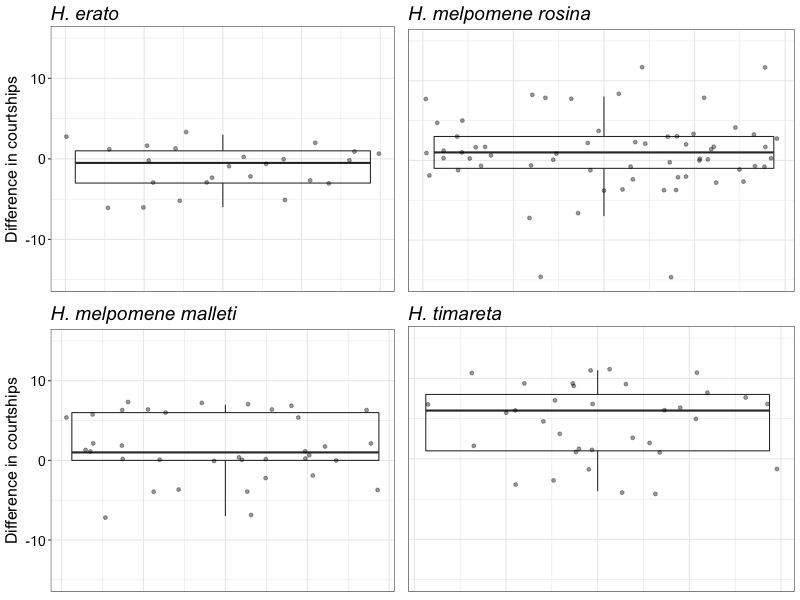


Figure S4: Proportion of courtships which resulted in different female behavioural responses. Males that mated are represented in green (left) and males that did not in orange (right). Means are marked with a black square and boxplots mark the inter-quartile ranges. Size of datapoint is proportional to the number of courtships by that male. Towards males which did not mate, *H. melpomene malleti* females were more likely to open their (2ΔlnL= 18.65, d.f.=1, p<0.001), fly away (2ΔlnL= 20.454, d.f.=1, p<0.01) and flutter (2ΔlnL= 32.303, d.f.=1, p<0.001). *H. timareta florencia* females were also more likely to open their wings (2ΔlnL= 24.875, d.f.=1, p<0.001), fly away (2ΔlnL= 12.3, d.f.=1, p<0.001), and flutter (2ΔlnL= 23.318, d.f.=1, p<0.001). No significant behavioural differences for *H. melpomene rosina* or *H. erato demophoon* were detected.
